# WEALTH AND HEALTH IN PREDICTING ELDERS’ SOCIAL CAPABILITIES IN CHINA: MEDIATING ROLE OF SOCIAL NETWORK

**DOI:** 10.1093/geroni/igz038.1348

**Published:** 2019-11-08

**Authors:** Yalu Zhang, Qin Gao, Fuhua Zhai, Paul Anand

**Affiliations:** 1 Columbia University, New York, New York, United States; 2 Fordham University, New York, New York, United States; 3 The Open University, Milton Keynes, United Kingdom

## Abstract

Despite an established positive link between social wealth, health, and social capability among older adults, the effect and mechanisms among these factors are understudied. This paper uses the WHO Study on Global Ageing and Adult Health (SAGE) data and a mediation analysis method, combing social capital theory and a social capabilities approach, to provide new evidence on the effects of financial resource, physical function, and cognitive function on the social capabilities of older adults (aged 55 and above) in China and the possible mediating role of social network in this relationship. The descriptive analysis results show that urban older adults (n=5,274), on average, had lower freedom of expression, lower sense of living safety, and less frequent community participation, while having better self-perceived health, higher physical and cognitive functions, more household income, and higher educational background than their rural peers (n=5,270). The Baron and Kenny’s mediation analysis results show that social networks accounted for a substantial proportion of the effects of wealth and health on social capabilities, but wealth and health still had strong, positive direct effects of its own. Higher mediating effects of social networks were found in the association between functions and social capabilities of freedom of expression (9.46%) and sense of safety (36.33%) among rural older adults. Results of this study urge for further social policies and intervention programs to enhance older adults’ social capabilities, including social cohesion, sense of trust and safety, physical and mental functioning, and subjective well-being.

